# Antimicrobial Edible Films and Coatings for Meat and Meat Products Preservation

**DOI:** 10.1155/2014/248935

**Published:** 2014-06-24

**Authors:** Irais Sánchez-Ortega, Blanca E. García-Almendárez, Eva María Santos-López, Aldo Amaro-Reyes, J. Eleazar Barboza-Corona, Carlos Regalado

**Affiliations:** ^1^DIPA, PROPAC, Facultad de Química, Universidad Autónoma de Querétaro, 76010 Querétaro, QRO, Mexico; ^2^Área Académica de Química, Instituto de Ciencias Básicas e Ingeniería, Universidad Autónoma del Estado de Hidalgo, Ciudad del Conocimiento, Carr. Pachuca-Tulancingo Km 4.5 Col Carboneras, 42184 Mineral de la Reforma, HGO, Mexico; ^3^División Ciencias de la Vida, Universidad de Guanajuato, Campus Irapuato-Salamanca, 36500 Irapuato, GTO, Mexico

## Abstract

Animal origin foods are widely distributed and consumed around the world due to their high nutrients availability but may also provide a suitable environment for growth of pathogenic and spoilage microorganisms. Nowadays consumers demand high quality food with an extended shelf life without chemical additives. Edible films and coatings (EFC) added with natural antimicrobials are a promising preservation technology for raw and processed meats because they provide good barrier against spoilage and pathogenic microorganisms. This review gathers updated research reported over the last ten years related to antimicrobial EFC applied to meat and meat products. In addition, the films gas barrier properties contribute to extended shelf life because physicochemical changes, such as color, texture, and moisture, may be significantly minimized. The effectiveness showed by different types of antimicrobial EFC depends on meat source, polymer used, film barrier properties, target microorganism, antimicrobial substance properties, and storage conditions. The perspective of this technology includes tailoring of coating procedures to meet industry requirements and shelf life increase of meat and meat products to ensure quality and safety without changes in sensory characteristics.

## 1. Introduction

Animal origin foods (AOF) constitute a good nutrients source for human diet, where their protein provides high biological value and essential amino acids which complement the quality of cereals and other vegetable proteins [1]. However, AOF are susceptible to chemical deterioration and microbiological spoilage and therefore represent a high risk for consumer health, in addition to producer economic losses. According to the Centers for Disease Control (CDC), every year foodborne illnesses account for about 48 million cases, 3,000 deaths, and 128,000 hospitalizations, reaching US $77.7 billion economic burden in the United States. In addition, reduced consumer confidence, recall losses, or litigation costs should be met by the food industry, whereas public health agencies pay the cost of responding to illnesses and outbreaks [2]. Losses can be greater in countries where less stringent regulation system and sanitary control is practiced. Outbreaks involving AOF comprise 40% of total US reported cases [3]. The presence of foodborne pathogens in a country food supply not only affects the health of local population but also represents a potential for pathogens spread by tourists and consumers where these food products are exported [4].

Edible coatings are food grade suspensions which may be delivered by spraying, spreading, or dipping, which upon drying form a clear thin layer over the food surface. Coatings are a particular form of films directly applied to the surface of materials and are regarded as part of the final product [5]. On the other hand, edible films are obtained from food grade filmogenic suspensions that are usually cast over an inert surface, which after drying can be placed in contact with food surfaces. Films can form pouches, wraps, capsules, bags, or casings through further processing and one of the main differences between films and coatings is their thickness.

The use of films in foods dates back to the 12th century in China where waxes were used to coat citric fruits to retard water loss, whereas the first edible film used for food preservation was made in the 15th century from soymilk (Yuba) in Japan. In England lard or fats were used as coating to prolong shelf life of meat products in the 16th century and in Europe; this process was known as “larding” [6, 7]. In the nineteenth century, a US patent was issued in relation to preservation of meat products by gelatin coatings [7, 8].

Edible films and coatings (EFC) are an alternative to extend the shelf life of AOF by acting as barriers to water vapor, oxygen, and carbon dioxide and as carriers of substances to inhibit pathogenic and spoilage microorganisms. Natural antimicrobial agents may be incorporated into the corresponding suspensions, adding functionality to edible films and coatings, leading to the antimicrobial edible films and coatings (AEFC) obtaining.

There is increased interest in development and use of AEFC to preserve meat quality for longer shelf life periods while maintaining food safety, which is based on consumers demand for natural and safe products. Industry is concerned about these issues, while keeping competitive production costs [9]. Other key issues are sustainability through the use of biodegradable packaging materials and applications of by-products from the food industry that can generate added value [5].

Due to similar properties of edible films and coatings this review discusses characteristics of both types of coverings applied to meat products. This work focuses on a critical discussion of issues raised by recent research findings on the effectiveness of antimicrobial films and coatings and their potential application to enhance safety and quality of meat products.

## 2. Meat Products

### 2.1. Meat Importance and Consumption

Meat (including poultry and fish) is the first-choice source of animal protein for many people all over the world [10]. According to the Codex Alimentarius [11] meat is defined as “all parts of an animal that are intended for, or have been judged as safe and suitable for human consumption.” Worldwide meat production is expected to be >250 million tons in 2014, with pork as the main product (108.9 million tons), whereas poultry production is expected around 87 million tons. Fish and seafood is also an important market, since world production in 2008 reached 142,287 tons [12]. Meat industry represents a significant share of national economies and therefore production and marketing systems should follow meat sanitation practices and additionally emerging preservation technologies such as the AEFC to extend shelf life and to avoid economic losses.

Nutritionally, meat importance is derived from its high quality protein containing all essential amino acids and its highly bioavailable minerals and vitamins [13]. In 2010, the average annual red meat consumption per capita in developing countries was 32.4 kg, whereas in industrialized countries was 79.2 kg, increasing to 124 kg/capita in the USA. Global annual poultry consumption rose from 11.1 to 13.6 kg/capita between 2000 and 2012. In 2009, fish accounted for 16.6% of world population intake of AOF and 6.5% of all protein consumed [13].

### 2.2. Meat Spoilage

Meat quality is highly dependent on preslaughter handling of livestock and postslaughter handling of meat [10]. Among the main factors affecting meat quality is pH, which is determined by the glycogen content of the muscle and varies from 5.4 to 5.7 in postrigor muscle; another important factor is temperature, which must be quickly decreased from 37°C to refrigeration temperatures (4–8°C) [14].

There are three mechanisms involved in meat and meat products deterioration during processing and storage: microbial spoilage, lipid oxidation, and enzymatic autolysis. Microbial population may arrive from native microflora of the intestinal tract and skin of the animals or through environmental, human, handling, and storage conditions associated to the production chain [15]. Microbial growth in meat can result in slime formation, structural components degradation, decrease in water holding capacity, off odors, and texture and appearance changes [10]. Lipid oxidation depends on fatty acids composition, vitamin E concentration, and prooxidants such as free iron in muscles. Oxidation products, such as hydroperoxides, aldehydes, and ketones, can cause loss of color and nutritive value due to degradation of lipids, pigments, proteins, carbohydrates, and vitamins [10, 16]. Enzymatic autolysis of carbohydrates, fats, and proteins of the tissues results in softening and greenish discoloration of meat and may lead to microbial decomposition. Proteolytic enzymes are active even at low temperatures (5°C) leading to microbial growth, loss of water holding capacity, and biogenic amines production [17].

### 2.3. Meat Related Outbreaks

Meat products outbreaks are often due to inadequate cooking or cross-contamination from other foods. However, contamination may occur while meat is processed, cut, packaged, transported, sold, or handled. Pathogenic microorganisms do not survive thorough meat cooking, but several of their toxins and spores do [10].

Red meat is frequently involved in outbreaks, mainly due to the presence of* Salmonella* spp.,* Listeria* spp.,* Clostridium* spp., and* Staphylococcus* spp. [3]. Most outbreaks reported in the EU in 2010 were due to meat and meat products consumption in which* Salmonella* was the main pathogen involved [18].* Listeria* infection is often considered as the most lethal; for instance, in 1998 hot dogs consumption caused 21 deaths and >100 illnesses [3]. Recently, an outbreak of* Salmonella typhimurium* was linked to the consumption of ground beef which caused hospitalization of seven people [19].

In the case of poultry* Salmonella* and* Campylobacter* account for most of the cases of food poisoning associated with chicken [3, 20]. In 2010 turkey contaminated with* Clostridium perfringens* caused 135 illnesses in Kansas (USA), whereas in 2011 ground turkey contaminated with* Salmonella* Heidelberg infected 136 people in 34 USA states [3].

Most outbreaks caused by fish and fish products are caused by natural toxins (scombrotoxin and ciguatoxin), rather than by bacteria or viruses. However, outbreaks caused mainly by* Vibrio parahaemolyticus* and* V. cholerae* in raw oysters have been reported; additionally* Clostridium botulinum*,* Staphylococcus aureus*,* Salmonella enterica*, and* Escherichia coli* were also involved in illnesses due to fish consumption [3, 21].

## 3. Edible Films and Coating Types

EFC act as barrier between food and the surrounding environment to enhance the quality of food products protecting them from physical, chemical, and biological deterioration. Design and application of EFC on meat products arises from the search of new preservation methods, the need to add value to by-products from renewable sources, the desire to give food products a more natural or ecological image, and reduction of environmental impact of using oil-derived plastic packaging materials [22]. Additionally, they may provide moisture loss reduction during storage of fresh or frozen meats, prevention of juice dripping, and decrease in myoglobin oxidation of red meats. There are two commercially available edible films, New Gem*™*, which contains spices and bilayer protein films that are used to enhance ham glaze and Coffi*™*, that is made from collagen nettings used to wrap boneless meat products [23]. Antimicrobials or antioxidant compounds incorporated into the polymer matrix may prevent growth of spoilage and pathogenic microorganisms, delay of meat fat rancidity, discoloration prevention, and even improvement of the nutritional quality of coated foods [24, 25].

### 3.1. Composition and Properties of Lipid-Based Films and Coatings

A wide range of hydrophobic compounds has been used to produce EFC, including animal and vegetable oils and fats (peanut, coconut, palm, cocoa, lard, butter, fatty acids, and mono-, di-, and triglycerides), waxes (candelilla, carnauba, beeswax, jojoba, and paraffin), natural resins (chicle, guarana, and olibanum), essential oils and extracts (camphor, mint, and citrus fruits essential oils), and emulsifiers and surface active agents (lecithin, fatty alcohols, and fatty acids) [26]. In meat products, emulsifiers and surface active agents are sometimes used as gas and moisture barriers. However, pure lipids can be combined with hydrocolloids such as protein, starch, cellulose, and their derivatives providing a multicomponent system able to be applied as meat coatings [27]. In fresh and processed meats, lipid incorporation into EFC can improve hydrophobicity, cohesiveness, and flexibility, making excellent moisture barriers, leading to prolongation of freshness, color, aroma, tenderness, and microbiological stability [24].

Palmitoylated alginate is the only lipid-containing material of AEFC recently reported to wrap beef muscle and ground beef [28] (Table 1). However, essential oil extracts have been widely used to promote antimicrobial activity of AEFC (column 3, Tables 1–3).

### 3.2. Composition and Properties of Protein-Based Films and Coatings

Film-forming proteins are derived from animals (casein, whey protein concentrate and isolate, collagen, gelatin, and egg albumin) or plant sources (corn, soybean, wheat, cottonseed, peanut, and rice). Protein-based films adhere well to the meat hydrophilic surfaces and provide barrier for oxygen and carbon dioxide but do not resist water diffusion [27]. Plasticizers, such as polyethylene glycol or glycerol, are added to improve flexibility of the protein network, whereas water permeability can be overcome by adding hydrophobic materials such as beeswax or oils like oleic that can affect films properties such as crystallinity, hydrophobicity, surface charge, and molecular size, improving films characteristics and their application [6, 31, 34]. Despite their advantages, protein films may be susceptible to proteolytic enzymes present in meat products or allergenic protein fractions may cause adverse reactions to susceptible people [24].

### 3.3. Composition and Properties of Polysaccharides-Based Film and Coatings

Polysaccharide coatings are generally poor moisture barriers, but they have selective permeability to O_2_ and CO_2_ and resistance to fats and oils [25]. Polysaccharide films can be made of cellulose, starch (native and modified), pectins, seaweed extracts (alginates, carrageenan, and agar), gums (acacia, tragacanth, and guar), pullulan, and chitosan. These compounds impart hardness, crispness, compactness, viscosity, adhesiveness, and gel-forming ability to a variety of films [24, 44, 45]. Polysaccharide films and coatings can be used to extend the shelf life of muscle foods by preventing dehydration, oxidative rancidity, and surface browning. When applied to wrapped meat products and exposed to smoke and steam, the polysaccharide film actually dissolves and becomes integrated into the meat surface resulting in higher yields, improved structure and texture, and reduced moisture loss [27].

Materials recently used to obtain AEFC in meat and meat products, poultry, and fish and fish products are shown in column 2 of Tables 1, 2, and 3, respectively. Chitosan based AEFC were the most commonly reported in recent years and have been used to wrap pork meat hamburgers and sausages [39, 42] (Table 1), as films and coatings on roasted and sliced turkey [46, 47] (Table 2) and as films on cod fillets [48] (Table 3). Chitosan was used as both polymeric material and antimicrobial agent, for roast beef coating [32] (Table 1) and chicken breast fillets (wrapping and coating) [49, 50] (Table 2) and as coating of Atlantic cod and herring [51] and as films on sea bass fillets [52] (Table 3). WPI and cellulose (or its acetate salt), despite being less reported, are also materials used in AEFC for meat and meat products [29, 30, 33, 35] (Table 1): turkey frankfurters [53] (Table 2) and smoked salmon [54] (Table 3). Several reports mention pectin for production of AEFC to wrap cooked ham and bologna [40] (Table 1) and chicken breast [55, 56] (Table 2), whereas other reports show gelatin based antimicrobial films placed on top or between slices of fish products [48, 57–59] (Table 3).

## 4. Common Antimicrobials Used in EFC

Incorporation of antimicrobial compounds into EFC as an alternative to their direct application onto the meat surface has the advantage of gradual release of the antimicrobial compound from the AEFC leading to a reduction of added antimicrobial and to reduced sensory changes. Antimicrobial compounds within AEFC are less exposed to interaction with meat surface components than those added directly to the surface and thus maintaining their activity [66–68].

Antimicrobial agents recently incorporated in AEFC for meat and meat products, poultry, and fish and fish products are shown in column 3 of Tables 1, 2, and 3, respectively. Target microorganisms aimed by recently developed AEFC as well as inoculation technique for meat and meat products, poultry, and fish and fish products are shown in columns 4 and 5 of Tables 1, 2, and 3, respectively.

The characteristics and mode of action of most common antimicrobials used to promote meat safety are described below.

### 4.1. Organic Acids

The antimicrobial effect of organic acids depends on concentration of undissociated form, which can penetrate the bacterial cell membrane. Inside the cell, their dissociation leads to interference with membrane transport and disruption of proton motive force [30]. Organic acids incorporated into EFC include lactate and acetate [46], propionate [18], and* p*-aminobenzoic acid [30]. WPI coatings added with malic acid, nisin, and grape seed extract applied on turkey frankfurters decreased to 2.3 log CFU/g of* L. monocytogenes* and 5 log CFU/g* S. typhimurium* after 28 d of storage at 4°C [53] (Table 1). Zein based AEFC using calcium propionate combined with nisin, reduced up to 5 log CFU/g of* L. monocytogenes* after 14 d at 4°C, when used to coat chicken breast [18]. Sodium lactate combined with other commercial antimicrobials reduced to 3.5 log/cm^2^ of this pathogen when roasted turkey was stored at 4°C for 8 weeks [46] (Table 2). Thus, organic acids, especially when acting combined with other antimicrobial agents, have an important role in maintaining microbiological quality of meat and meat products.

### 4.2. Essential Oils and Plant Extracts

Essential oils are complex mixtures of volatile compounds obtained from plants, which mainly include terpenes, terpenoids, and aliphatic chemicals, all characterized by low molecular weight [69]. Oils containing phenols such as thymol, carvacrol, and eugenol exhibit the highest activity against all kind of microorganisms. Essential oils usually show higher antibacterial activity than mixtures of their major antimicrobial components, suggesting that minor components are critical for enhanced activity [69]. The antimicrobial mechanism is attributed to the disturbance of the cytoplasmic membrane disrupting the proton motive force; active transport and coagulation of cell contents may occur [70]. Direct incorporation of essential oils in the formulation of AEFC applied to meat products is expected to reduce bacterial population but may alter their sensory characteristics [68]. Microencapsulation of essential oils or their ingredients may be an alternative to protect them from interaction with environmental factors, avoiding their oxidation or volatilization while exerting their antimicrobial effect. Moreover, encapsulation increases the oil solubility in water, prevents its release at an undesired stage, and makes it easier to handle [71, 72]. Essential oils or their constituents that may be incorporated in AEFC on AOF include those extracted from lemongrass, oregano, pimento, thyme, or cinnamon [40, 57, 65]. Oregano essential oil has been the most commonly reported in recent years including a 1.5% extract (v/v), successfully used to reduce total viable count by 2 log CFU/g of cold smoked sardine covered with an AEFC after 20 d storage at 5°C [58], whereas at 1.9% it achieved* L. monocytogenes* population reduction by 2.4 log CFU/g after 28 d, at 4°C in wrapped cold smoked salmon [65] (Table 3). Oregano essential oil combined with thyme extract, was incorporated into a film placed on top and bottom of fresh ground beef patties reducing* Pseudomonas* spp. and coliforms populations [36], whereas mixed with pimento essential oil, the films covering beef muscle slices reduced to 1 log of* E. coli* O157:H7 after 7 d of storage at 4°C [31] (Table 1). Grapefruit seed extract (GSE) incorporated into AEFC was found to inhibit* E. coli* O157:H7 and* L. monocytogenes* from pork loins [34], bacon [41], and salmon [59] (Tables 1 and 3). However, some commercial GSE is adulterated with synthetic preservatives such as benzalkonium and benzethonium chlorides, which are solely responsible for the antimicrobial activity of GSE. These compounds show toxicity and allergenicity to humans, and it is unlikely that they are formed during any extraction and/or processing of grapefruit seeds and pulp [73, 74].

### 4.3. Bacteriocins

Bacteriocins from lactic acid bacteria are peptides produced by bacteria that inhibit or kill other related and unrelated microorganisms [75]. These agents are generally heat-stable, apparently hypoallergenic and readily degraded by proteolytic enzymes in the human intestinal tract [68]. Class I bacteriocins, such as nisin, bind to plasma membranes via nonspecific electrostatic interactions and have a dual mode of action. The antibacterial activity results from pore formation in the bacterial plasma membrane, leading to dissipation of the transmembrane potential and vital solute gradients. The high efficiency of pore formation is the result of a second mechanism involving the cell wall precursor Lipid II which increases the affinity of nisin for the membrane, stabilizes a transmembrane orientation of nisin, and forms and integral part of the nisin pore. The pore structure involves a complex made up of four lipid II and 8 nisin molecules, which interferes with peptidoglycan biosynthesis [76, 77]. Other bacteriocins such as pediocin have been widely studied in food systems, but nisin remains the only one approved by European Union (EU) and the USA where it enjoys GRAS status [68, 78]. The effect of nisin incorporation into AEFC is the most studied, either to protect beef and turkey frankfurters, or turkey bologna against* L. monocytogenes* [33, 53, 61] (Tables 1 and 2); but pediocin has also been tested [35].

### 4.4. Proteins

Lysozyme is a naturally produced enzyme active against gram-positive bacteria, by hydrolyzing N-glycosidic bonds connecting N-acetyl muramic acid with the fourth carbon atom of N-acetyl glucosamine of the peptidoglycan molecule in the cell wall. This antimicrobial has been formulated in whey protein isolate (WPI) films and tested for its diffusivity and antimicrobial effect on salmon slices [79] and also tested in ground beef patties using zein films [38] (Table 1).

### 4.5. Chitosan

Chitosan is a linear polysaccharide composed of randomly distributed *β*-(1-4)-linked D-glucosamine and N-acetyl-D-glucosamine. Chitosan is believed to chelate certain ions from the lipopolysaccharide (LPS) layer of the outer membrane of bacteria or to exhibit electrostatic interactions among its NH_3_^+^ groups and the negative charges of microbial cell membrane. In both cases cell permeability increases releasing key cellular components of bacteria. The antimicrobial action of chitosan is influenced by type of chitosan, degree of polymerization, and environmental conditions. Chitosan coatings act as barrier against oxygen transfer leading to growth inhibition of aerobic bacteria [42]. In addition to the functionality of chitosan as polymeric material and antimicrobial agent (Section 3.3), it has been used as coating and wrapper in salami [37] and as film and coating combined with lauric arginate and nisin to reduce* L. monocytogenes* population in sliced turkey deli meat [47] (Tables 1 and 2) and also in seafood and fish [48, 52].

### 4.6. Lauric Arginate

Lauric arginate (LAE) is a food-grade cationic surfactant that is highly active against a wide range of food pathogens and spoilage microorganisms including bacteria, yeasts, and molds. It is obtained through the reaction of L-arginine, hydrochloric acid, ethanol, thionyl chloride, sodium hydroxide, lauryl chloride, and deionized water [80]. LAE affects cells viability by disturbing membrane potential and causing structural changes, although no disruption of cells is detected. In gram-negative cells, LAE alter both the cytoplasm membrane and the external membrane, while in gram-positive cells, alterations were observed in the cell membrane and in the cytoplasm. However, in both cases, cells remained intact and cell lysis is not observed [81]. LAE is nontoxic and is metabolized to naturally occurring amino acids, mainly arginine and ornithine, after consumption. Effectiveness of LAE, alone or in combination with other antimicrobials, has been tested against* L. monocytogenes*,* S. enterica*, and* L. innocua* in cooked ham and sliced turkey deli meat producing 2 log reductions in all cases [43, 47] (Tables 1 and 2).

Antimicrobial agents recently incorporated in AEFC for meat and meat products, poultry, and fish and fish products are shown in column 3 of Tables 1, 2, and 3, respectively, whereas application conditions and effect of AEFC are shown in columns 6 and 7 of the same tables, respectively.

## 5. Migration of Antimicrobial Agents from Films

Few reports have considered the migration extent of antimicrobial agents from edible films to the food surface. A study showed the effect of film thickness, solution pH, and temperature on nisin migration from an active WPI edible film to an aqueous solution. Results indicated that nisin is able to migrate from the film where diffusivity increased at lower pH and thickness, while it increased at higher temperatures [82]. Sorbic acid migration from an active cellulose film into pastry dough was evaluated for 40 days and it was not significantly affected by film thickness, achieving a migration of 0.07%, (w/v) [83]. Nisin release measured from low density polyethylene film was unpredictable but it was affected by temperature and pH [84]. Migration of lysozyme from WPI-glycerol films indicated that the diffusion coefficient decreased as the WPI-glycerol ratio increased or storage temperature decreased [79]. Chitosan-glycerol films incorporated with 1–10% (w/v) lauric arginate showed full release of the agent and followed a Fickian behavior in a few hours at 4° and 28°C. Films were active in liquid and solid media against bacteria, yeast and fungi achieving 1.8–5.8 log reductions [50]. These findings lead us to consider that antimicrobial agents incorporated into AEFC may prevent microbial contamination of food surfaces.

## 6. Application and Effect of AEFC on Meat Products

Antimicrobial packaging can be a promising tool for protecting meat from pathogens contamination by preventing microbial growth by direct contact of the package with its surface. The gradual release of an antimicrobial substance from a packaging film to the food surface for extended period of time may be more advantageous than incorporating the antimicrobial into foods [85].

Studies using chitosan films incorporated in meat products demonstrated that lipid oxidation is reduced, suggesting that it may be due to the antioxidant activity of chitosan [52], as well as its low oxygen permeability characteristic [42]. Similar results have been obtained when other compounds were incorporated such as essential oils [57], grapefruit extracts [41, 59], and lysozyme [38]. In all cases, the oxidation rates decreased maintaining an acceptable quality in meat, poultry, or fish products. However, even when the coating may confer protection against lipid oxidation, other characteristics may have changed, leading to modified sensory attributes that made the food unacceptable for consumers. Application of films on meat surface in some cases could increase the stability of the red meat color [57], but if coatings act as gas barriers undesirable color changes may occur [38]. Sensory studies on fish indicated that not only bacterial number is critical for fish acceptance, but other factors such as bacterial types, autolytic activity, biochemical properties of fish, and storage conditions are significant [76]. In other studies, using chitosan film incorporated with oregano essential oil did not negatively influence the taste of chicken samples, extending the shelf-life of chicken fillets by 14 days, maintaining acceptable sensory characteristics [49]. Therefore, each particular application should be evaluated to establish the conditions leading to maintain meat safety without altering sensory characteristics.

Potential benefits of using AEFC for the meat industry are prevention of moisture loss, avoiding texture, flavor, and color changes, producing a significant economic impact by increasing saleable weight of products. Other advantages include reduction of dripping enhancing products presentation and reduced use of absorbent pads at the bottom of trays. Low oxygen permeability leads to decreased lipids oxidation and brown color-causing myoglobin oxidation, reduced load of spoilage and pathogenic microorganisms, and partial inactivation of deteriorative proteolytic enzymes at the surface of coated meat. Volatile flavor loss and foreign odors pick-up by meat, poultry, or seafood could be restricted by using edible films and coatings and incorporation of additives such as antimicrobial agents can be used for direct treatment of meat surface. There are, however, some factors that may represent disadvantages of using AEFC; there is wide diversity of meat products whose characteristics may vary making it difficult to standardize a single application procedure. Composition and properties of AEFC will provide different functionality and may affect scaling up of application methods for coatings.

Selection of the appropriate AEFC for a specific meat product will depend on its nature, characteristics, specific needs, costs, and benefits that this technology can offer to the manufacturers and the consumer. Thus, more research is needed to improve production and application processes of AEFC intended for the meat industry to be economically feasible and appropriate for each product.

## 7. Conclusions

The application and effects of AEFC of different nature have been investigated in several AOF. Effectiveness shown by each one depends on meat source, polymer used, film barrier properties, target microorganism, antimicrobial substance, and conditions of storage among others. EFC are a good alternative to improve the quality and safety of food and also to add value to food industry by-products. However, some challenges remain such as the need to improve and standardize coating procedures according to industry requirements aiming to reduce costs and increase shelf life to meet consumer demands without altering sensory characteristics of meat and meat products.

## Figures and Tables

**Table 1:** Use of antimicrobial films and coatings in meat and meat products.

Product	Coating material	Antimicrobial compound	Target microorganism	Inoculation technique	Conditions	Results	Reference
Sliced bologna and summer sausage	Whey protein isolate (WPI, pH 5.2) films	0.5 to 1.0% *p*-aminobenzoic acid (PABA) and/or sorbic acid (SA)	*L.monocytogenes, E. coli* O157:H7, and *S. enterica* typhimurium (10^6^ CFU/g)	0.1 mL of inoculum spread onto both surfaces	4°C, 21 days (d) Slices placed in plates covered with edible film Stored in aerobiosis	WPI films with SA or PABA reduced *L. monocytogenes, E. coli, *and* S. typhimurium* populations by 3.4–4.1, 3.1–3.6, and 3.1–4.1 log CFU/g, respectively, on both products	[29]

Hot dogs (beef 60%, pork 40%)	Whey protein isolate films (casings)	*p*-aminobenzoic acid (PABA) 1%	*L. monocytogenes* (10^3^ CFU/g)	Immersion in *L. monocytogenes* culture for 1 min and dried in a safety cabinet	4°C, 42 d Vacuum-packaged samples	Growth inhibition for 42 d in refrigeration but no population reduction. Controls increased around 2.5 CFU/g	[30]

Beef muscle slices	Milk protein films	Oregano essential oil (OR) 1.0% (w/v), Pimento essential oil (PI) 1.0% (w/v), or 1% OR-PI (1 : 1)	*Escherichia coli* O157:H7 or *Pseudomonas* spp. (10^3^ CFU/cm^2^)	Spreading over meat surface Samples placed in plates, covered on either side with the corresponding film	4°C, 7 d Meat sterilized by radiation and then inoculated Samples in plates were hermetically sealed	Film with OR was the most effective against both bacteria Reduction of 0.95 log of *Pseudomonas* spp. and 1.12 log reduction of *E. coli* O157:H7	[31]

Sterile beef muscle slices or ground beef	Palmitoylated alginate films Activated alginate beads	Covalently immobilized nisin (N) to activated alginate beads (AAB) (0–1000 IU/mL), or ground beef mixed with 0–1000 IU/mL of N	*Staphylococcus aureus* (10^4^ CFU/g)	Inoculated using a sterile spoon and placed in sterile plates	4°C, 14 d Covered with immobilized nisin film or mixed with nisin solution	Reduction of 0.91 and 1.86 log CFU/cm^2^ on samples covered with film (500 or 1000 IU/mL, resp.) After 14 days: N solution (500 or 1000 IU/g) mixed with ground beef reduced to 2.2 and 2.81 log CFU/g, respectively; N (500 or 1000 IU/g) in AAB reduced to 1.77 and 1.93 log CFU/g, respectively	[28]

Roast beef	Chitosan (CH, high or low molecular weight) coatings dissolved in lactic or acetic acid	Chitosan, lactic, or acetic acid (0.5 and 1%; w/v)	*Listeria monocytogenes *(10^6^ CFU/g)	1 mL culture onto 5 g cubed meat, air dried 10 min Then dipped in chitosan for 30 s, dried for 1 h and placed into sterile Whirl-Pack bags	4°C, 28 d 5 g cubed roast beef samples placed into sterile bags	Reduction of 1–3 log CFU/g for low molecular weight chitosan in acetic and lactic acids, respectively, after 28 d	[32]

Frankfurters	Cellulose (produced by *G. xylinus*) films	Nisin (N), 625 and 2500 IU/mL	*L. monocytogenes *Scott A serotype 4b (10^6^ CFU/mL)	Dipping in 0.85% saline sln. containing *L. monocytogenes* for 2 s	4°C, 14 d Samples wrapped in a single layer of film and vacuum-sealed for 2.5 s	Films containing 625 IU/mL N not significantly reduced *L. monocytogenes* populations. Films with 2500 IU/mL N decreased 2 log CFU/g compared to the control	[33]

Pork loins	*Gelidium corneum*–gelatin (GCG) films	Grapefruit seed extract (GFSE, 0.08% w/v) or green tea extract (GTE, 2.80% w/v)	*E. coli *O157:H7 (NCTC12079) and* L. monocytogenes *(KCTC 3710) (10^5^ CFU/g each one)	Spread with a sterile glass rod and allowed to drain for 10 min	4°C, 10 d Samples were packed in direct contact to films and stored in sterile polystyrene trays	Samples packed with the GCG film containing GFSE or GTE decreased population of *E. coli* O157:H7 and *L. monocytogenes* in 1 and 2 log CFU/g, respectively, compared to the control	[34]

Ham	Cellulose acetate films	Pediocin (ALTA 2351) (25% and 50%, w/v)	*L. innocua* (10^6^ CFU/mL) and *Salmonella* sp. (10^6^ CFU/mL)	Immersion in a 0.1% w/v peptone solution of *L. innocua *or* Salmonella* sp. for 10 min	12°C, 15 d Samples sterilized under UV (5 min each side) Films intercalated with ham slices packed in plastic bags and vacuum sealed	The 50% pediocin-film reduced *L. innocua* 2 log relative to the control. The 25% and 50% pediocin-films had similar performance on *Salmonella *sp. inhibition, both presenting 0.5 log reduction relative to the control	[35]

Fresh ground beef patties	Soy protein films	Oregano (OR), thyme (TH), or OR-TH essential oils (5%)	*E. coli *O157:H7,* Staphylococcus aureus, Pseudomonas aeruginosa, and Lactobacillus plantarum *	No inoculation	4°C, 12 d Film applied to the upper and bottom surfaces of patties and vacuum packaged in plastic bags	*Pseudomonas *spp. in samples coated with TH and OR films decreased in 1.13 and 1.27 log CFU/g, respectively. Coliforms were reduced by 1.6, 1.9 and 2.0 log CFU/g with addition of OR, OR-TH, and TH, respectively	[36]

Salami	Sodium caseinate (SC) films and coatings	Chitosan (CH) (2%)	Mesophilic and psychrotrophic aerobic bacteria and yeast and mold	No inoculation	10°C, 5 d, 65% RH. Film added by immersion and as wrapper Immersed slices air dried at 30°C and 50% RH for 50 min All food faces were contacted with wrapping film	CH and SC/CH films applied as both, coatings and wrappers, exerted a strong bactericidal action on 3 microbial populations analyzed, with reductions of 2 to 4.5 log CFU/g	[37]

Ground beef patties	Zein films	Lysozyme (LY) (43 mg/g) and disodium Ethylene diamine tetra acetic acid (Na_2_EDTA, 19 mg/g)	Mesophilic microorganisms (TVC) and coliforms (TCC)	No inoculation	4°C, 7 d Films at both sides of each piece Wrapped with stretch plastic film and with aluminum foil	After 5 and 7 d, TVC of patties with LY and Na_2_EDTA films were significantly lower (0.75–1.9 log CFU/g) than control films. After 5 d, TCC of patties with LY and Na_2_EDTA films were significantly lower than the control but after 7 d, no significant difference in TCC of patties was found	[38]

Pork meat hamburgers	High molecular weight chitosan (1% w/v), acetic acid (1% w/v), lactic acid (1% w/v) films	Sunflower oil (1%)	Mesophilic bacteria, coliforms	No inoculation	5°C, 8 d Surface of both sides of the hamburgers coated with the films placed in PET trays	Reduction of 0.5–1 log for mesophilic microorganisms; 1 log CFU/g for coliforms	[39]

Cooked ham and bologna	High methoxyl pectin + Apple, carrot or hibiscus puree films	Carvacrol (CV) or cinnamaldehyde (CM) (0.5%, 1.5%, and 3.0%, w/v)	*L. monocytogenes* (101M; serotype 4b) 10^6^ CFU/mL	Dispersed on the surface as droplets. Inoculated samples dried under the biohood (30 min), flipped over, and inoculated on the other side Inoculated samples were dried again (30 min) and then surface wrapped with one of the test films	4°C, 7 d Samples were kept frozen and thawed before use. Sample wrapped in 2 pieces of circular films and parts of the films were not directly in contact with the meat surface	Films containing 3% CV showed 3 log reductions on ham at day 7. Bologna, films with 3% CV reduced 2 log CFU/g at day 7. Reductions with 1.5% CV were 0.5–1, 1–1.5, and 1-2 logs at day 0, 3, and 7, respectively. Films containing 3% CM, only 0.5–1.5 and 0.5–1.0 log CFU/g reductions were seen at day 7 on ham and bologna, respectively. Limited reduction (0.2-0.3 log CFU/g) was observed with 1.5% CM films	[40]

Bacon	Red algae (RA) films	1% w/v, grapefruit seed extract (GFSE)	*Escherichia coli *O157:H7 (10^6^ CFU/g) and* L. monocytogenes *(10^7^ CFU/g)	Spread separately on the surface of bacon with a sterile glass rod and allowed to rest for 30 min	4°C, 15 d Packed by wrapping	*E. coli* O157:H7 decreased 0.45 log CFU/g and *L. monocytogenes* decreased by 0.76 log CFU/g respect to the controls	[41]

Pork sausages	Chitosan films	Green tea extract 20% (w/v) in the chitosan film-forming solution	Mesophilic bacteria, yeasts and molds, lactic acid bacteria (LAB), not inoculated	No inoculation	4°C, 20 d Sausages wrapped with films, packaged into a pouch of low density polyethylene coated with polyamide plastic bag and heat-sealed	On day 12, faster growth in control samples for total viable count and molds and yeast was found; no difference for LAB	[42]

Cooked cured ham	Polylactic acid (PLA) films	Lauric arginate (LAE) (0% to 2.6%, w/w)	*Listeria monocytogenes* and *Salmonella enterica* serovar typhimurium (10^5^ CFU/mL)	Inoculum of both *L. monocytogenes *and *S. typhimurium* onto surface of the sliced ham	4°C, 7 d Slices sterilized with UV on each side prior to inoculation Inoculated samples wrapped with LAE-coated PLA film and stored in closed plates	LAE-coated PLA film (2.6%) showed a significantly greater antibacterial activity, with *L. monocytogenes *and* S.typhimurium* levels reduced to <2 log CFU/film after 24 h exposure and remaining at this low level for the next 6 d	[43]

**Table 2:** Use of antimicrobial films and coatings in poultry.

Product	Coating material	Antimicrobial compound	Target microorganism	Inoculation technique	Conditions	Results	Ref.
Chicken breast (ready-to-eat cooked chicken cubes)	Zein coatings dissolved in propylene glycol (ZP) or ethanol (ZE)	Nisin (N) (1000 IU/g) and/or calcium (CP) propionate (1% w/v)	*L. monocytogenes* V7 (low and high inoculum 2.67 log CFU/g and 6.89 log CFU/g, respectively, at 4°C)	Cubes immersed in 24 h broth cultures for 30 s, allowed to drip free of excess inoculum, and dried. Frozen samples were irradiated (3.0 kGy) and kept frozen until used	4°C or 8 °C, 24 d. Cubes boiled in water bath for 20 min, were inoculated, then dried, followed by dipping in edible ZP or ZE, with and without antimicrobials. Air dried samples (20 min) stored in sterile bags	*L. monocytogenes* was reduced by 4.5–5 log CFU/g relative to the control after high dose and 16 d at 4°C, with more significant effect when N was added to the films. Low inoculum dose and using ZPNCP film caused complete inhibition from 4 to 24 d, either at 4° or at 8°C	[18]

Turkey frankfurter	WPI coatings	Grape seed extract (GSE, 1.0–3.0% w/v), nisin (N, 6–18 kIU/g), malic acid (MA 1.0–3.0%; w/v), EDTA (1.6 mg/mL), and their combinations	*L. monocytogenes, E. coli* O157:H7, and *Salmonella typhimurium* (10^6^ CFU/g)	Samples were defrosted and dipped into cultures of 10^6^ CFU/mL of *L. monocytogenes, E. coli *O157:H7, or *S. typhimurium *for 1 min at room temperature. Inoculated samples were then air dried under laminar flow conditions	4°C, 28 d Samples were dipped in film-forming solutions (1 min) and dried (10 min, room temperature). Samples were then packed individually in sterile bags, and stored	*L. monocytogenes* decreased to 2.3 log/g (N, 6000 IU/g; GSE 0.5%; MA 1.0%). *S. typhimurium* decreased to 5 log CFU/g using any antimicrobial, whereas *E. coli* decreased to 4.6 log cycles using N, MA and EDTA. All reductions were relative to the control	[53]

Chicken breast	High methoxyl pectin 11400 with apple puree films	Carvacrol (C) or cinnamaldehyde (CM) at 0.5–3% (w/w).	*Salmonella enterica *serovar *Enteritidis* or* E. coli *O157:H7 (ATCC 35150) (10^7^ CFU/g)	Inoculum was dispersed on the surface as droplets	23°C or 4°C, 3 d Samples were dipped in boiling water (40 s), plated and exposed in a bio-hood for drying (30 min). Sample was flipped over and inoculated in a similar way. Meat was wrapped using appropriate edible films	At 23 °C, films with 3% antimicrobials showed the highest reductions (4.3–6.8 log CFU/g) of both *S. *Enteritidis and *E. coli *O157:H7. At 4°C, C exhibited greater activity than CM. Relative to control samples, films with 0.5–3% C reduced *S. *Enteritidis by 1.6–3 log CFU/g, whereas 1–3% CM films reduced its population by 1.2–2.8 log CFU/g. Films with 0.5–3% C reduced 1–3 log *E. coli* whereas 1–3% CM films inhibited 0.2–1.2 log CFU. Treatments were at 4°C	[55]

Chicken breast	*k*-carrageenan (*k*CF) films	Ovotransferrin, OTf (25 mg), EDTA (5 mM), and potassium sorbate (PS, 10 mg/g of *k*-carrageenan)	*E. coli *	No inoculation	5°C, 7 d Samples were wrapped with *k*-carrageenan-based films and packed in plastic bags	Samples wrapped with *k*CF added with 5 mM EDTA alone or mixed with 25 mg OTf allowed 2.7 log CFU/g reduction of *E. coli*, compared to the control, at day 7. Addition of 25 mg of OTf or 10 mg PS slightly inhibited microbial growth	[60]

Turkey bologna	Gelatin films	Nisaplin based films (GNF) (0.025–0.5 %; w/v nisin) and Guardian CS1-50 based films (GGF) (0.5–4 %; w/v).	*L. monocytogenes *(10^6^ cfu/mL)	Inoculated by surface spreading. Samples were thawed at 4°C for 18 h and then inoculated and covered with antimicrobial film. Each sample was vacuum-sealed	4°C, 56 d Samples were irradiated at 4°C (2.4 mrad for 521 min) and stored at −70°C	Both 0.5% GNF and 1% GGF inhibited *L. monocytogenes *by 4 log CFU/cm^2^ and 3 log CFU/cm^2^, respectively, relative to the control, during storage at 4°C for 56 d. GGF inhibited *L. monocytogenes* by 2.17 log CFU/cm^2^ at 7 d	[61]

Roasted turkey	Starch, chitosan, alginate, or pectin coatings	Sodium lactate (SL) and sodium diacetate (SD). OptiForm PD4 (OF4), NovaGARDCB1 (NG1), Protect-M (PM), and Guardian NR100 (GN)	A cocktail of five strains of *L. monocytogenes *(PSU1 serotype 1/2a, F5069 serotype 4b, ATCC19115 (serotype 4b), PSU9 serotype 1/2b, and Scott A serotype 4b) 10^3^ UFC/mL	Spreading on both sides of the turkey surface, 10^3^ CFU/cm^2^. After inoculation, turkey samples were kept at 4°C for 20 min	4°C, 8 weeks Coatings on each side were dried in a laminar-flow hood for 20 min each side. All samples were inserted into nylon/polyethylene pouches and vacuum sealed	OF4 (2.5%) alone or mixed with PM (0.12%) in films made from alginate, chitosan or pectin were the most effective, reaching *L. monocytogenes* reduction by 3.5 log/cm^2^ relative to the control after storage	[46]

Chicken breasts	3% solution of high methoxyl pectin added with golden delicious apple puree films	Carvacrol (C) and cinnamaldehyde (CM) (0.5–3.0 %; w/v)	*Campylobacter jejuni* (D28a, H2a and A24a), 10^7^ CFU/mL	Samples dipped in boiling water for 40 s and dried in a biohood for 1 h. Chicken was dip-inoculated for 5 min	23°C and 4°C, 72 h in anaerobiosis. Samples placed in sterile plates, dried in a 42°C, 10% CO_2_ incubator for 1 h and then wrapped with apple films and stored	Films with ≥1.5% CM reduced populations of both strains to below detection at 23°C at 72 h. Films with 3% C reduced populations of A24a and H2a to below detection. Using 3% C, films reduced to 0.5 log CFU/g of both strains A24a and D28a and 0.9 logs for H2a at 4°C	[56]

Chicken breast fillets	Chitosan (CH) coating, deacetylation degree of 75–85%	Chitosan (1.5 % w/v) and/or oregano oil 0.25% v/w (OO)	Mesophilic microorganisms, *Pseudomonas *spp. and *Brochothrix thermosphacta *	No inoculation	12, 18 y 21 d, 4°C Samples were dipped into the chitosan solution (1.5 min) and drained. Sterile OO was added to the surface. Fillets were packed in plastic pouches, and stored in a modified atmosphere	Shelf life of chicken fillets can be extended using either OO and/or CH, by approximately 6–21 d	[49]

Chicken breasts fillets	Chitosan (CH) films	CH or CH-LAE (1, 5 or 10%,by weight)	Mesophiles, psychrophiles, yeast, moulds, *Pseudomonas*, coliforms, LAB, and hydrogen sulfide-producing bacteria	No inoculation	0, 2, 6 and 8 d, 4°C Slices wrapped with CH or CH-5% LAE films, then packed in polyethylene films	CH films reduced 0.47–2.96 log population of fillets, depending on time and microbial group studied. Incorporation of LAE (5%) increased antimicrobial activity to 1.78–5.81 log reduction, and maintaining the initially low microbial fillets load for 8 d	[50]

Sliced turkey deli meat	Chitosan (CH, 2–5% w/w) films and coatings added with 2% solution of either acetic, lactic or levulinic acids	Lauric arginate (LAE, 50–200 mL/mL) and nisin (NIS, 25 mg/mL) alone or in combination	*L. innocua *(6-7 log CFU/cm^2^)	Even spread over the meat surface (3 × 3 cm^2^) using sterile spreaders	48 h, 37°C. Films were placed on top of inoculated turkey; coatings applied by spreading. The product was vacuum packed and stored at 10°C for 24 h prior to microbiological analysis	High CH levels reduced 4.6 log CFU/cm^2^. NIS addition (486 IU/cm^2^) reduced *Listeria* by 2 and 2.4 log CFU/cm^2^ for 2% and 5% CH, respectively. Combination of CH, LAE and NIS had similar reductions as only CH with LAE, suggesting no additive or synergistic effect by NIS. Despite no statistical difference (*P* < 0.05), coatings showed more microbial reduction than films	[47]

**Table 3:** Use of antimicrobial films and coatings in fish and seafood.

Product	Coating material	Antimicrobial compound	Target microorganism	Inoculation technique	Conditions	Results	Ref.
Atlantic cod (*Gadus morhua*) and herring (*Clupea harengus*)	Chitosan coatings	Chitosan (CH) with different molecular weights and viscosities (14, 57 or 360 mPa s)	Psychrotrophic microorganisms (PT) and total plate count (TPC)	No inoculation	12 d, 4°C Samples immersed in CH solution (5°C) 30 s and after 2 min a second immersion for 30 s. Then they were dried at 40°C for 2 h in a forced air oven and stored	Herring fillets treated with 57 and 360 cP CH showed lower PT population than 14 cP CH-treated fillets after 6 d. CH treatments reduced to 10^3^ and 10^2^ TPC of herring and cod samples, respectively, after 12 d	[51]

Smoked salmon	Whey protein isolate (WPI) coatings	Lactoperoxidase system (LPO) (0–0.5%, w/v)	*L. monocytogenes *(V7 serotype 4b, LCDC 81-861 serotype 4b, Scott A serotype 4b, 101M and 108M) (10^2^–10^4^ CFU/g).	Spotted directly onto the salmon (I + C) or on top of applied coating (C + I) and spread with a hockey stick	4°C and 10°C, 35 d. I + C samples were inoculated and then dried for 0.5 h and coated. C + I samples dried for 1 h and then inoculated	Samples coated by LPO-WPI showed <1.0 log CFU/g of *L. monocytogenes* at 4°C for 35 d (both treatments). *L. monocytogenes* was completely inhibited in C + I samples stored during 35 d at 10°C	[54]

Cold-smoked sardine (*Sardina pilchardus*)	Gelatin (G) films	Oregano extract (OE) (*Origanum vulgare* 1.5%, v/v) or rosemary (RM) (*Rosmarinus officinalis*, 20% w/v) or Chitosan (CH) (1.5% w/v), high pressure (300 MPa/20°C/15 min) (HP).	Total viable count (TVC), H_2_S-reducing organisms, luminescent bacteria, and Enterobacteriaceae	No inoculation	5°C, 20 d Fish slices were placed between two layers of edible films and were stored in clean bags	Fish coated with OE-G and RM-G films reduced TVC by 1.99 and 1.54 log CFU/g respectively, on d 16. H_2_S-reducing bacteria followed a similar pattern. OE and RM had no effect, but CH reduced to ≤10^3^ CFU/g in all cases. Pressurized samples produced undetectable levels of all microorganisms for 20 d, except uncoated sample whose TVC was 10^5^ CFU/g at d 20	[58]

Cod (*Gadus morhua*)	Gelatin (G) or in combination with chitosan (CH) films	Clove essential oil (CO)	Total bacterial count (TVC), H_2_S-producers organisms, luminescent organisms, *Pseudomonas, *Enterobacteriaceae (EB), and lactic acid bacteria (LAB)	No inoculation	2°C, 11 d Fillets were covered with the G-CH film containing CO, and vacuum-packed in plastic bags	TVC count was 6.1 log CFU/g. Luminescent bacteria reached 6 log CFU/g after 3 d, but later were undetected. H_2_S producers were completely inhibited from d 3 onwards. LAB and EB increased during storage despite storage temperature	[48]

Cold smoked salmon (CSS) slices and fillets	Alginate (AL), *κ*-carrageenan, pectin, gelatin, or starch coatings	Sodium lactate (SL, 0–2.4% w/v) and sodium diacetate (SD, 0–0.25% w/v), OptiForm (OF, 2.5% w/v)	Mixture of* L. monocytogenes* strains: PSU1, PSU9, F5069, ATCC 19115, and Scott A. 10^3^ CFU/g	Surface-inoculated	4°C, 30 d Samples coated with AL incorporating SL/SD or OF, dried 20 min and stored at 4°C in vacuum sealed bags	Al coatings with 2.4% SL/0.25% SD and OF reduced *L. monocytogenes* by 3.2 and 4 log CFU/g (slices) and 2.4 and 3 log CFU/g (fillets), respectively, relative to control sample	[62]

Bream (*Megalobrama amblycephala*)	Alginate (AL) coatings	Vitamin C (VC, 5% w/v) and tea polyphenols (TP, 0.3% w/v)	TVC	No inoculation	21 d, 4°C Bream was dipped in AL-antimicrobial solutions (1 min), air-dried (1 min) and immersed in 2% (w/v) CaCl_2_ (1 min) to obtain gels. Samples were packed and stored	After 4 d of storage. The TVC of VC and TP decreased by 1.6 and 1.5 log CFU/g, respectively, on day 21	[63]

Sea bass slices	Gelatin extracted from the skin of unicorn leatherjacket (*Aluterus monoceros*) films	Lemongrass essential oil (LEO) 25% (w/w)	Mesophilic (TVC) and psychrophilic (PS) microorganisms, enterobacteria (EB), and H_2_S-producing bacteria LAB	No inoculation	12 d, 4°C For each slice, films were placed on both sides. Subsequently, the samples were placed in polystyrene trays wrapped with extensible polypropylene film	TVC of unwrapped sample increased to 7.2 log CFU/g at d 4 reaching 7.9 log CFU/g at d 12. TVC of LEO-film wrapped samples was 5.6 log CFU/g at d 12. PS count for control, G and LEO films was 6.0, 5.5 and 4.0 log CFU/g, respectively. LAB increased to 7.2, 6.7 and 5.9 log CFU/g at the end of storage. LEO-film showed the lowest EB counts (2.2 log CFU/g), as compared to control	[57]

Sea bass (*Dicentrarchus labrax*)	Chitosan (CH) films	CH with vacuum packaging	Total mesophilic aerobic bacteria (TVC) and psychrotrophic (PS) aerobic bacteria	No inoculation	4°C until end of shelf life. Fish fillets were covered using CH films, wrapped and vacuum packaged using polyethylene bags	The acceptable limit of 6 log CFU/g and 7 log CFU/g for PS and TVC bacteria, respectively, was reached after 25 d at 4°C. Control samples reached this limit after 5 d	[52]

Indian oil sardine (*Sardinella longiceps*)	Chitosan (CH) (1 and 2% w/v) coatings	Chitosan (1 and 2% w/v)	Mesophilic microorganisms (TVC)	No inoculation	11 d, 1-2°C. Fillets were dipped in 1 and 2% CH at 1-2°C for 10 min, drained for 5 min and placed in trays for 24 h, then sealed using HDPE	Eating quality was maintained for 8 and 10 d for 1 and 2% CH respectively, whereas untreated samples lasted 5 d. The limit of 10^7^ CFU/g of TVC was exceeded after 7, 9 and 11 d for untreated, 1% and 2% CH treated samples, respectively	[64]

Salmon	Barley bran protein and gelatin (BBG) films	Grapefruit seed extract (GFSE) (0.5–1.2% w/v)	*E. coli* O157:H7 and *L. monocytogenes *(10^6^ CFU/mL)	*E. coli* O157:H7 and *L. monocytogenes *were spread individually on sample surface and allowed to rest for 30 min	4°C, 15 d Samples wrapped using the BBG film. Samples packed in polyethylene terephthalate film were used as control	After 15 d, populations of *E. coli* and *L. monocytogenes* inoculated salmon with the BBG film containing GSE decreased by 0.53 and 0.50 log CFU/g, respectively, compared to the control	[59]

Cold-smoked salmon	Potato processing waste (PPW) films	Oregano essential oil (OO) 0.97% and 1.92% (185 and 289 mg oil/g film)	*L. monocytogenes* V7 (6.7–6.9 log CFU/g)	*L. monocytogenes* Overnight culture (100 *μ*L) was spotted at 25–30 locations on salmon fillet, spread and dried in a biological hood (30 min)	4°C, 28 d Salmon samples were wrapped with edible films and were vacuum packed	Coated samples with PPW-OO, reduced *Listeria* population by 0.4–2.4 log CFU/g as compared to control samples, after storage period	[65]
